# Data Descriptor: Daily observations of stable isotope ratios of rainfall in the tropics

**DOI:** 10.1038/s41598-019-50973-9

**Published:** 2019-10-08

**Authors:** Niels C. Munksgaard, Naoyuki Kurita, Ricardo Sánchez-Murillo, Nasir Ahmed, Luis Araguas, Dagnachew L. Balachew, Michael I. Bird, Supriyo Chakraborty, Nguyen Kien Chinh, Kim M. Cobb, Shelby A. Ellis, Germain Esquivel-Hernández, Samuel Y. Ganyaglo, Jing Gao, Didier Gastmans, Kudzai F. Kaseke, Seifu Kebede, Marcelo R. Morales, Moritz Mueller, Seng Chee Poh, Vinícius dos Santos, He Shaoneng, Lixin Wang, Hugo Yacobaccio, Costijn Zwart

**Affiliations:** 1 College of Science and Engineering, James Cook University, Cairns, Australia; 2 Research Institute for Environment and Livelihoods, Charles Darwin University, Darwin, Australia; 3 Institute for Space-Earth Environmental Research (ISEE), Nagoya University, Nagoya, Japan; 4 Stable Isotopes Research Group and Water Resources Management Laboratory, Universidad Nacional, Heredia, Costa Rica; 5 Isotope Hydrology Division, Institute of Nuclear Science and Technology, Bangladesh Atomic Energy Commission, Dhaka, Bangladesh; 6 Isotope Hydrology Section, International Atomic Energy Agency, Vienna, Austria; 7 Center for Climate Change Research, Indian Institute of Tropical Meteorology, Pune, India; 8 Department of Isotope Hydrology, Center for Nuclear Techniques, Ho Chi Minh City, Vietnam; 9 School of Earth and Atmospheric Sciences, Georgia Institute of Technology, Atlanta, USA; 10 Isotope Hydrology Laboratory, National Nuclear Research Institute, Accra, Ghana; 11 Institute of Tibetan Plateau Research, Chinese Academy of Sciences, Beijing, China; 12 Environmental Studies Center, São Paulo State University, Butanta, Brazil; 13 Department of Earth Sciences, Indiana University-Purdue University Indianapolis (IUPUI), Indianapolis, USA; 14 Earth Research Institute, University of California Santa Barbara, Santa Barbara, California USA; 15 Department of Earth Sciences, Addis Ababa University, Addis Ababa, Ethiopia; 16 Inst. de Biodiversidad y Biología Experimental y Aplicada, Universidad de Buenos Aires, Buenos Aires, Argentina; 17 Faculty of Engineering, Computing & Science, Swinburne University of Technology, Kuching, Malaysia; 18 School of Marine and Environmental Sciences, Universiti Malaysia Terengganu, Terengganu, Malaysia; 19 Earth Observatory of Singapore, Nanyang Technological University, Singapore, Singapore

**Keywords:** Atmospheric dynamics, Palaeoclimate, Hydrology

## Abstract

We present precipitation isotope data (δ^2^H and δ^18^O values) from 19 stations across the tropics collected from 2012 to 2017 under the Coordinated Research Project F31004 sponsored by the International Atomic Energy Agency. Rainfall samples were collected daily and analysed for stable isotopic ratios of oxygen and hydrogen by participating laboratories following a common analytical framework. We also calculated daily mean stratiform rainfall area fractions around each station over an area of 5° x 5° longitude/latitude based on TRMM/GPM satellite data. Isotope time series, along with information on rainfall amount and stratiform/convective proportions provide a valuable tool for rainfall characterisation and to improve the ability of isotope-enabled Global Circulation Models to predict variability and availability of inputs to fresh water resources across the tropics.

## Introduction

### Background & Summary

This database is an outcome of the International Atomic Energy Agency’s (IAEA) coordinated Research Project (CRP) F31004 on ‘Stable isotopes in precipitation and paleoclimatic archives in tropical areas to improve regional hydrological and climatic impact models’. The project was conducted from 2012–2017 with participation from the following member States: Argentina, Australia, Bangladesh, Brazil, China, Costa Rica, Ethiopia, Ghana, India, Japan, Singapore, United States of America (USA) and Vietnam.

The stable isotopes of water (i.e., ^18^O/^16^O and ^2^H/^1^H, expressed as δ^18^O and δ^2^H values hereafter) are effective integrating tracers of regional-scale hydroclimate processes^1,2^. The key objective of the CRP was to improve understanding of the links between stable isotopes in precipitation and the hydroclimatic factors controlling them in tropical regions from daily to annual timescales and site to regional spatial scales. To achieve this objective the CRP initiative collected daily precipitation samples for analysis of stable isotope ratios of oxygen and hydrogen across the tropics. Several sampling stations with no prior rainfall isotope observations were established. It is noteworthy that this data set is the first systematic effort to capture ground-based daily isotope record of tropical rainfall in all phases (i.e. neutral, warm, and cool) of the El Niño-Southern Oscillation (ENSO). Furthermore, the 2015–2016 ENSO event was one of the strongest on record^3^.

The availability of Global Circulation Models (GCMs) incorporating isotope physics provided a promising framework to study isotopic variability in precipitation^4–8^. To evaluate the stable isotope fields simulated by such GCMs, the IAEA’s Global Network of Isotopes in Precipitation (GNIP) database^9,10^ has recurrently been used as the major comprehensive source of data. While isotope-enabled GCMs simulate modern isotopic patterns in global precipitation on monthly and inter-annual time scales reasonably well over mid and high latitude regions, simulations of tropical rainfall need further development^6,8^. A major reason for this problem is that the physical mechanisms controlling the spatial and temporal isotopic variations in tropical precipitation are still not fully understood. In addition, the poor spatial and temporal coverage of precipitation isotope data in tropical regions poses a challenge to explore the primary drivers of isotope variability. Therefore, an expanded monitoring network in the tropics is required, not only to identify the key controls of isotopic variability, but to improve the reproducibility of GCMs simulations. This improvement may help elucidate the mechanisms (e.g. sub-cloud evaporation, moisture convergence and entrainment) that control isotopic changes in precipitation and enhance the capabilities of climate models to predict variability and availability of fresh water resources. In addition, better understanding of the controls on precipitation isotope variability will lead to improvements in our interpretation of isotope-based proxies in terrestrial and maritime paleoarchives (e.g., caves, corals, lake sediments, and potentially tree-rings)^11^.

In the tropics, precipitation isotopes mainly correlate negatively with precipitation amounts on a monthly scale. This empirical low-latitude inverse correlation between water isotope ratios and the amount of rainfall, known as the ‘amount effect'^12^, has been used as a rationale to infer wet and dry paleo-hydroclimate periods based on available maritime and terrestrial paleo proxies across the tropics^13^. However, the amount effect is not universal over the tropical regions. For example, in SE Asia the effect is relatively strong at two marine island stations (Palau and Bali) but relatively weak at continental coastal stations (e.g., Bangkok and Da Nang)^14^. Furthermore, the correlation between precipitation amount and isotopic composition is commonly weaker or non-existent on a daily basis compared to monthly time scales^2,14,15^. This is because the linear regression approach neglects other processes such as moisture convergence and entrainment, resulting in weak correlations across the tropics with >80% of the variance unexplained when using daily data, whereas stronger correlations (30–70% variance explained) are reported when computing monthly means^2^. These complexities indicate that the isotopic variations in precipitation, even at tropical islands where a pronounced amount-effect is observed, are not directly controlled by rainfall amount but is rather influenced by the other convection related processes (e.g. cloud microphysics, cloud type, moisture transport). This conclusion is supported by several recent studies which have demonstrated that the isotopic variability is associated with regional, rather than local, convective activity (e.g. in North Africa^16^, Tibet^17^, Borneo^1^, Pacific Ocean^18,19^, India^20,21^, Australia^22^, Brazil^23^ and Costa Rica^15^). In Costa Rica, isotopic variations also exhibited more complex interactions between variable moisture sources, humidity and orographic lifting^15^. A gradual decrease in δ^18^O values of precipitation with cumulative rainfall along upstream air mass trajectories over several days was reported in Tibet^17^, Northern Australia^22^, and Southeast Asia^24^. On the other hand, in some amount-effect-dominated regions, changes in moisture source and air mass trajectories largely influenced sub-monthly or seasonal isotopic variations (e.g. East Africa^25,26^, Japan^27^, Namibia^28^).

It is well known that intra-seasonal isotopic variations are clearly seen in many tropical regions (e.g. Borneo^1^, Western Pacific^19^, Northern Australia^22^). These variations typically manifest as negative excursions in δ^18^O and δ^2^H values and typically, but not exclusively, correspond to the wet phases of the Madden Julian Oscillation (MJO). Cyclonic lows (i.e., hurricanes, typhoons) may also produce extreme rainfall and negative δ^18^O and δ^2^H anomalies. Within the MJO wet phase, large organised convective cloud systems, referred to as mesoscale convective systems (MCSs), account for a large portion of tropical rainfall. Stratiform rainfall associated with MCSs has been shown to be mainly associated with large negative excursions of δ^18^O and δ^2^H values in tropical rainfall^19,29^. Negative isotope anomalies in stratiform rainfall have been linked to deposition of ^18^O- and ^2^H-depleted water vapour onto ice particles at altitude which, as they fall, aggregate and melt at mid tropospheric levels^29,30^. Most recently, the relative influence of bulk precipitation microphysics, cloud type, and surface moisture transport on precipitation amounts and ^18^O/^16^O ratios was assessed in the tropics^2^. This analysis showed that bulk precipitation microphysics and cloud type (i.e., stratiform rain fraction) exert comparable influences on the isotopic composition of precipitation, whereas moisture transport plays an important secondary role in regions of deep atmospheric convection. Reduced Outgoing Longwave Radiation (OLR) values are indicative of stronger MCS activity and often correlate with low δ^18^O values in rainfall^20,22^. Based on these results, we can hypothesise that stratiform rainfall is a major driver of isotopic variability over the amount-effect dominated region. However, currently both spatial and temporal coverage of precipitation isotope data is insufficient to adequately test the hypothesis.

Here we present stable isotope data for daily precipitation collected at nineteen stations at both maritime and continental locations within the tropics; ten stations are (near-) coastal and nine are located from ≈ 80 to 600 km inland. We also calculated daily mean stratiform rainfall area fractions at these stations using TRMM and Global Precipitation Mapping (GPM) satellite observations.

## Methods

### Sampling and analysis

**Figure 1:**
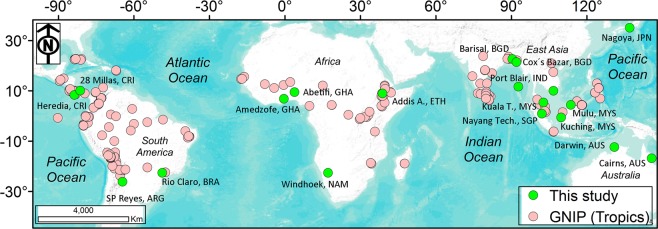
Map of the 19 sampling stations (green dots) and 229 GNIP (Global Network of Isotopes in Precipitation) tropical stations (pink dots; ranging from 23.76°N/23.83°S and 90.30°W/125.26°E). Geographical coordinates for stations of this study are provided in Table 1.

Details of the sampling stations including location, updated Köppen-Geiger climate zone classification^31^, mean annual precipitation and temperature, sampling period and number of samples collected are provided in Table 1 and Fig. 1. Rainfall samples were usually collected at 9am local time but variations of up to a few hours occurred on some occasions for practical reasons.

**Table 1:** Location and climate information for rainfall sampling stations.

Contributing country	Station	Köppen-Geiger climate classification^31^	Sampling period	Number of observations	Latitude (degrees)	Longitude (degrees)	Altitude (m asl)	Marine (M) or land (L) dominated	Mean annual P (mm)	Mean annual T (°C)
Argentina	SP Reyes, Argentina	Bsh/Bsk	2014–15	30	24.14 S	65.39 W	1400	L	556	15.9
Australia	Cairns, Australia	Am	2014–17	405	16.82 S	145.68E	27	M	2386	25.0
Australia	Darwin, Australia	Aw	2014–17	252	12.36 S	130.89E	5	M	1694	27.7
Bangladesh	Barisal, Bangladesh	Aw	2013–15	234	22.72 N	90.35E	7	M	2068	25.9
Bangladesh	Cox’s Bazar, Bangladesh	Am	2015	104	21.44 N	91.97E	8	M	4713	25.6
Brazil	Rio Claro, Brazil	Cfa	2014–17	254	23.40 S	47.54 W	632	L	1294*	20.3*
Costa Rica	28 Millas, Costa Rica	Af	2014–17	582	10.10 N	83.37 W	18	M	3032	22.3
Costa Rica	Heredia, Costa Rica	Aw	2013–17	440	10.00 N	84.11 W	1150	M	2554	20.9
Ethiopia	Addis Ababa, Ethiopia	Cwb	2014	135	9.00 N	38.76E	2440	L	1143*	16.3*
Ghana	Abetifi, Ghana	Af	2014–15	83	6.68 N	0.63 W	595	M	1566*	22.6*
Ghana	Amedzofe, Ghana	Af	2014–16	95	6.85 N	0.43 W	686	M	1350*	27.0*
India	Port Blair, India	Am	2012–16	558	11.66 N	92.73E	16	M	3068*	26.4*
Japan	Nagoya, Japan	Cfa	2013–17	399	35.15 N	136.97E	137	M	1632	16.4
Singapore	Nanyang Tech, Singapore	Af	2013–16	469	1.35 N	103.68E	42	M	2378*	26.8*
Singapore	Kuching, Malaysia	Af	2014–16	295	1.46 N	119.41E	5	M	4093*	26.9*
Singapore	Kuala Terengganu, Malaysia	Af	2014–16	206	5.41 N	103.09E	5	M	2761*	26.8*
USA	Mulu, Malaysia	Af	2013–17	1091	4.05 N	114.81E	32	M	3839*	27.0*
USA	Windhoek, Namibia	Bwh	2012–15	109	22.61 S	17.10E	1721	L	359*	19.5*
Vietnam	HCM City, Vietnam	Am	2013–15	331	10.04 N	106.69E	5	M	1868*	27.4*

*Data from Climate-data.org where not supplied by site investigator.

Table 2 provides sampling, laboratory and instrumental details along with the analytical precision claimed by the individual laboratories. Most stations used the IAEA-designed rain collector^32^ (Palmex, Zagreb, Croatia) which minimises secondary evaporation from the sample.

**Table 2:** Investigator, sampling and analytical information.

Contributing country /Chief Investigator	Sampling method	Laboratory	Instrument	δ^2^H precision ‰ (1σ)	δ^18^O precision ‰ (1σ)
Argentina/H.D. Yacobaccio	Pluviometer	INGEIS	LGR DLT-100	0.5	0.2
Australia/N.C. Munksgaard	IAEA rain collector	James Cook University & Charles Darwin University	Picarro L2120-i, L2130-i (diffusion sampler)	0.5	0.1
Bangladesh/N. Ahmed	IAEA rain collector	INST and IAEA hydrology	LGR LWIA-24-EP	1.32	0.22
Brazil/D. Gastmans	IAEA rain collector	IGCE/UNESP	LGR LWIA-24-EP, T-LWIA-45-EP	1.2	0.2
Costa Rica/R. Sánchez-Murillo	IAEA rain collector	Stable Isotopes Research Group, Universidad Nacional de Costa Rica	Picarro L2120-i	0.5	0.1
Ethiopia/S.Kebede	IAEA rain collector	IAEA/NERC-Keyworth, UK	Picarro L2120-i	0.8	0.1
Ghana/S. Ganyaglo	IAEA rain collector	IAEA hydrology/GAEC	LGR DLT-100	1.0	0.2
India/S. Chakraborty	IAEA rain collector/rain gauge	Indian Institute of Tropical Meteorology	LGR TIWA-45-EP	1.0	0.1
Japan/N. Kurita	Rain gauge	Nagoya University	Picarro L1102-i	1.0	0.1
Singapore/S. He	IAEA rain collector	EOS, Nanyang Technical University	Picarro L2130-I, L2140-i	0.5	0.1
USA/K. M. Cobb	Copper rain gauge	Georgia Institute of Technology	Picarro L2130-i	0.5	0.1
USA/L. Wang	Rain gauge	Indiana University-Purdue University Indianapolis Ecohydrology Lab	LGR TWVIA-45-EP	0.8	0.2
Vietnam/K.C. Nguyen	IAEA rain collector	Center for Nuclear Techniques	LGR DLT-100	1.0	0.15

All isotope data are reported as δ^2^H and δ^18^O values (in ‰) relative to the VSMOW/SLAP scale with δ_VSMOW_ defined as the zero point: δ = ((R_sample_-R_VSMOW_)-1)*1000 (‰), where R corresponds to the absolute isotope abundance ratios of ^2^H/^1^H and ^18^O/^16^O.

### Calculation of Stratiform Rainfall Fraction

**Figure 2:**
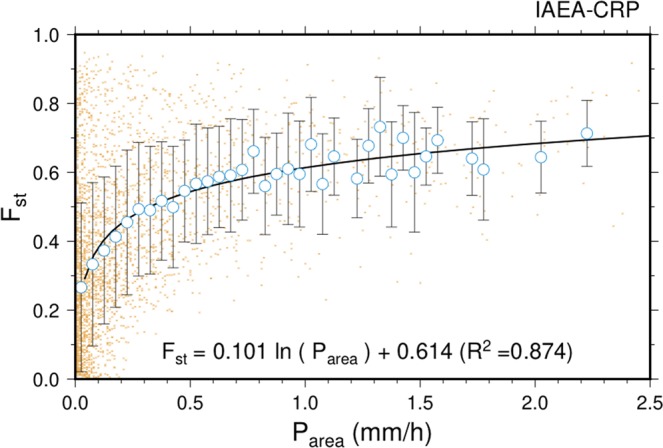
Relationship between stratiform rainfall area fraction (F_st_) and the area-averaged rainfall amount (P_area_) over the 5° × 5° longitude/latitude box centred on each station during the period where rainfall was sampled for isotopic analysis. Orange dots represent each individual data. Blue circles with error bars represent the average and standard deviation in precipitation intensity bins for each 0.05 mm/h interval up to 1.5 mm/h. Solid curved line shows a logarithmic regression of averaged values.

The daily mean stratiform rainfall area fraction (F_st_) was calculated to examine the influence of stratiform rainfall on the daily isotopic variability. F_st_ is defined as the average percent of rainfall area covered by stratiform rainfall over the 5° x 5° longitude/latitude box centered over each isotope monitoring station. We used the Ku-band Precipitation Radar (KuPR) convective/stratiform classification data from version 5, level 2 product of GPM (Global Precipitation Measurement) Core Observatory (https://pmm.nasa.gov/GPM), which is a successor of the TRMM Precipitation Radar. The GPM satellite flies at an altitude of 407 km in a non-sun-synchronous orbit and completes roughly 16 orbits per day between 65° N and 65° S. The KuPR’s horizontal footprint size along a track (swath width) is 245 km. Based on this swath width, 164 orbits are required to cover the whole equator (roughly 40,000 km) corresponding to around 10 days. This means that only a few GPM orbits pass through the domain (5° x 5° longitude/latitude box) within 10 days and so we cannot obtain daily convective/stratiform classification data from the satellite. As an alternative approach, we used area averaged (5° x 5° box) daily precipitation to estimate the daily F_st_. In the tropics, stratiform rainfall area is significantly larger than the convective rainfall area, and the increase in rain area is more likely to result in larger total rain amounts^33,34^. We found statistically significant relationships of area-averaged rainfall amount (P_area_) to F_st_ over the study domains at fourteen stations (i.e., Cairns, Darwin, 28 Millas, Heredia, Barisal, Cox´s Bazar, Port Blair, Nanyang Tech., Mulu, Ho Chi Minh City (HCM City), Kuala Terengganu., Kuching, Abetifi, and Amedzofe) from 2014 to 2017 (p < 0.05, R2 range = 0.25–0.72, mean R^2^ = 0.53). As shown in Fig. 2, combining data from the 14 stations show a strong correlation between P_area_ and F_st_ (R^2^ = 0.87, p < 0.01). However, at five inland or slightly extra-tropical stations (i.e., Rio Claro, Addis Ababa, Windhoek, Nagoya and SP Reyes) the relationships were weak, and we excluded these stations from further analysis. We then applied the correlation shown in Fig. 2 to a satellite-based daily precipitation dataset to estimate the daily F_st_ over the study domain at 14 stations. A Tropical Rainfall Measuring Mission (TRMM) Multi-satellite Precipitation Analysis product, namely TMPA 3B42, was used to estimate area-averaged daily precipitation at each station. The TMPA 3B42 product has a 3-hourly temporal resolution and a 0.25° spatial resolution^35^. Original data are available online at http://disc.gsfc.nasa.gov/datacollection/TRMM_3B42_V7.shtml.

**Figure 3:**
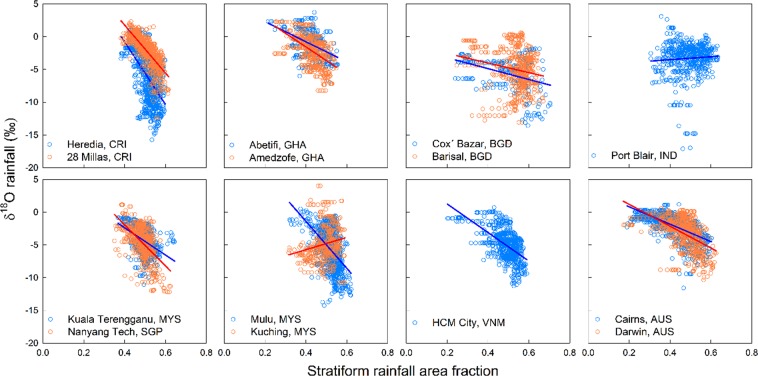
Relationship between10-day moving average of rainfall δ^18^O values and stratiform rainfall area fractions (5° × 5° box centered on each station) at 14 tropical stations. Refer to Table 1 for sampling period for each station and Table 3 for linear coefficients and correlation coefficient (R^2^).

**Figure 4:**
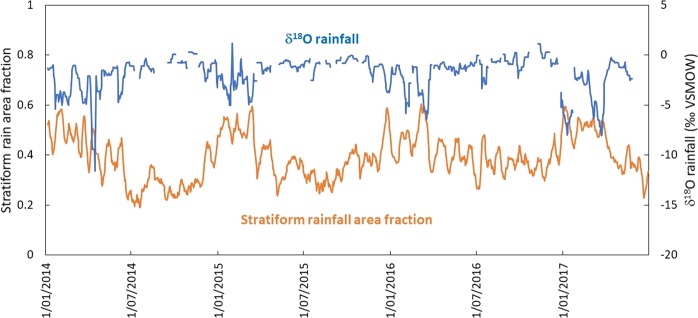
Cairns time series of 10-day moving average of rainfall δ^18^O values and stratiform rainfall area fractions (5° × 5° box centred on Cairns) from January 1, 2014 to July 1, 2017.

Figure 3 shows the correlation between the 10-day moving average rainfall isotope data and stratiform rainfall area fractions >0.01 for 14 tropical stations. Since there is a time lag for organized convective cloud systems (MCSs) in the upwind region to arrive at rainfall sampling stations, the moving average improves correlation compared to using the daily data at most stations. The stations Heredia and 28 Millas in Costa Rica, Darwin and Cairns in Australia, Amedzofe in Ghana, Nanyang Tech in Singapore, Mulu in Malaysia and HCM City in Vietnam had the strongest correlations (R^2^ = 0.28–0.58) while the remaining stations had weaker or insignificant correlations (p > 0.05) (Table 3). Figure 4 shows a time series for the 10-day moving averages of rainfall isotope data and stratiform rainfall area fraction at Cairns, where a relatively strong anti-phase variation of these two parameters (R^2^ = 0.43) is observed.

**Table 3:** Observations (N), correlation coefficients (R^2^) and linear coefficients of relationship between 10-day moving averages of rainfall δ^18^O value and stratiform rainfall area fraction (5° x 5° box centred on station). Statistically significant (p < 0.05) values are underlined.

Station	N	R^2^	slope	intercept
Cairns, Australia	1090	0.43	−13.3	+3.5
Darwin, Australia	747	0.28	−16.7	+4.5
Barisal, Bangladesh	586	0.04	−7.3	−1.1
Cox’s Bazar, Bangladesh	250	0.13	−8.3	−1.6
28 Millas, Costa Rica	796	0.58	−36.6	+16.4
Heredia, Costa Rica	929	0.32	−47.9	+18.4
Abetifi, Ghana	347	0.19	−15.4	+5.4
Amedzofe, Ghana	565	0.29	−20.9	+6.8
Port Blair, India	641	<0.01	+2.3	−4.4
Nanyang Tech, Singapore	624	0.32	−31.4	+10.6
Kuching, Malaysia	448	0.04	+9.4	−9.5
Kuala Terengganu, Malaysia	544	0.18	−20.8	+5.9
Mulu, Malaysia	1227	0.25	−35.9	+12.9
HCM City, Vietnam	739	0.36	−21.8	+5.6

## Technical Validation

All laboratory analyses were carried out using infrared laser-absorption spectrometry either by Off-Axis Integrated Cavity Output Spectroscopy (Los Gatos Research, San Jose, CA, USA) or by Cavity Ring Down Spectroscopy (Picarro, Santa Clara, CA, USA). These spectrometers can produce accurate and precise results provided that volatile organic compounds do not cause spectral interferences. However, such interferences are generally absent from rainfall samples and can easily be monitored using instrument software.

The datasets presented here were quality controlled by the individual investigators and laboratories. During the CRP participants were provided with guidance on the production, use and calibration of in-house (secondary) standard waters traceable to the International Measurement Standards VSMOW and SLAP (IAEA 2019) to ensure analytical accuracy and optimise quality control procedures. Most of the CRP’s laboratories participated in the IAEA Water Isotope Inter-Comparison WICO2016^36^ as well as an unofficial CRP-wide inter-comparison.

## Data Records

Data sets are available at figshare.com (https://figshare.com/s/fdfabb43a844cad530a5). The file ‘CRP isotopes’ contains stable isotope data in daily rainfall at 19 stations sampled within the period 2012 to 2017. They are recorded in the following order: Local sampling start date/time (YYYY-MM-DDTHH:MM), local sampling end date/time (YYYY-MM-DDTHH:MM), precipitation δ^18^O (‰ VSMOW), precipitation δ^2^H (‰ VSMOW), precipitation *d*-excess (‰ VSMOW), precipitation amount (mm). Note that at some stations only the sampling end date was recorded (sampling of 24-hr rainfall occurred at 9:00 local time). Empty cells indicate that no data was obtained.

The file ‘CRP stratiform P’ contains calculated stratiform precipitation area fraction for the subset of 14 stations for which this fraction could be calculated (five stations were excluded, see above). They are recorded in the following order: Local sampling start date/time (YYYY-MM-DDTHH:MM), local sampling end date/time (YYYY-MM-DDTHH:MM), sampled precipitation amount (mm), precipitation δ^2^H (‰ VSMOW), precipitation δ^18^O (‰ VSMOW), precipitation *d*-excess (‰ VSMOW), satellite observation date (YYYY-MM-DDT), daily mean area-averaged precipitation amount (mm), daily mean stratiform precipitation area-fraction, moving 10-day average precipitation δ^18^O (‰ VSMOW), moving 10-day average stratiform precipitation area-fraction. Stratiform rainfall area fractions <0.01 were disregarded due to their high uncertainty. Empty cells indicate that no data was obtained.

## Usage Notes

We encourage interested parties to contact the site investigators to explore possible collaboration opportunities based on these data. It is noted that some data have been published in peer-reviewed journals.
